# A randomised controlled trial assessing the effects of weather sensitivity profile and walking in nature on the psychophysiological response to stress in individuals with coronary artery disease. A study protocol

**DOI:** 10.1186/s40359-024-01574-3

**Published:** 2024-02-19

**Authors:** Dalia Martinaitienė, Francisco Sampaio, Zsolt Demetrovics, Biljana Gjoneska, Justina Portačenko, Austėja Damulevičiūtė, Toma Garbenytė-Apolinskienė, Julius Burkauskas, Nijolė Kažukauskienė

**Affiliations:** 1 Laboratory of Behavioral Medicine, Neuroscience Institute, Lithuanian University of Health Sciences, Vyduno al. 4, Palanga, LT-00135 Lithuania; 2 Nursing School of Porto, Rua Dr. António Bernardino de Almeida, 830, 844, 856, 4200-072 Porto, Portugal; 3 CINTESIS@RISE, Nursing School of Porto (ESEP), Rua Dr Plácido da Costa, 4200-450 Porto, Porto, Portugal; 4 Institute of Psychology, ELTE Eötvös Loránd University, Izabella Utca 46, Budapest, 1064 Hungary; 5 Centre of Excellence in Responsible Gaming, University of Gibraltar, Gibraltar, Gibraltar; 6 Macedonian Academy of Sciences and Arts, Skopje, North Macedonia

**Keywords:** Coronary artery diseases, Weather sensitivity, Cardiac rehabilitation, Nature, Stress reaction, Walking

## Abstract

**Background:**

The following protocol pertains to a pioneer study, aiming to investigate how weather sensitivity and walking in different environments affects the psychophysiological responses to the stress of individuals with coronary artery disease (CAD) during rehabilitation (WE_SENSE_THE_NATURE). This randomised control trial will provide fresh insight on the influence of the environmental exposure in CAD patients, as it is seldom investigated in association to the disease. Additionally, findings on the link between personality traits and cognitive functions (especially cognitive flexibility), and weather sensitivity may help reveal a fine-grained perspective on the treatment possibilities for individuals with CAD at risk to stress-vulnerability.

**Methods:**

The proposed protocol is for a randomised control trial among individuals attending a cardiac rehabilitation program. We aim to recruit 164 individuals, collecting information related to demographic characteristics, weather sensitivity, functional capacity, personality traits, subjective mental health status, cognitive function, and basal cortisol level of participating individuals. Basal cortisol level refers to cortisol concentration in saliva and will be tested in the morning and the afternoon prior to the day of the experiment. After baseline measurements, the patients will be randomly assigned to either walking outdoors or walking indoors. All measures and their sequential order will remain the same within each group, while the treatment condition (i.e., walking environment) will vary between groups. On the day of the experiment, hemodynamic parameters (assessed via 6-hour blood pressure measurements), stress level (consisting of assessments of cortisol level), and mood (assessed using visual analogues scale) will be registered. Cold stress test will be administered to evaluate the effect of walking in different environments.

**Discussion:**

The outcomes of this study may have direct clinical applications for the use of different types of exercise environments in cardiac rehabilitation programs. Awareness about the potential influence of weather sensitivity on the psychophysiological reactions to stress in individuals with CAD may contribute to a timely planning and implementation of actions leading to improved medical care services and preventive measures, especially considering the expected weather oscillations and extreme weather events due to unfolding of the climate change.

**Trial registration:**

This protocol has been retrospectively registered in ClinicalTrials.gov with identifier code: NCT06139705 on November 20, 2023.

**Supplementary Information:**

The online version contains supplementary material available at 10.1186/s40359-024-01574-3.

## Background

The environment might influence one’s course of a disease and health prospective through direct exposure to physical, chemical, social, and psychosocial risk factors, as well as indirectly, through behavior-related changes response to those factors [1]. Classical risk factors (pertaining to dietary and lifestyle habits) only partially account for variations in the occurrence, incidence, and mortality of cardiovascular disease (CVD). Therefore, other, less-explored factors need to be taken in consideration when referring to the epidemiology of the disease [2].

Most notably, stress (both physiological and psychological) affects the biological processes involved in the progression of coronary artery disease (CAD) [1]. Stress is associated with a wide range of diseases that involve almost every physiological system. Mounting experimental evidence exists on the link between stress and physiological or psychological functions of individuals, as well as the psychophysiological reactions to stressful and challenging environmental exposures [3–6].

In this regard, physical activity largely contributes to the prevention of CAD, improves the prognosis of patients, and decreases depressive symptoms [7, 8]. Indeed, the restorative effects of walking in nature have been documented and accumulating over the past 30 years, gaining lot of attention over the years [9]. Outdoor exercise, appears to be more beneficial than the same activity in urban settings or indoors, based on some experimental indications from psychological, physiological, and biochemical metrices [9–11]. Nevertheless, some studies and recent systematic reviews or meta-analyses [12, 13] suggest differences in the effects of natural versus urban environments. For example, Mygind and colleagues [14] show that seated relaxation and walking in natural environments has enhanced heart rate variability to a greater extent, than the same activities that were performed in control conditions, even though data about cortisol concentration levels remained inconsistent. In addition, some isolated findings suggest that even short-term visits to nature have a more pronounced effect on perceived stress relief, compared to the built environment, with no marked differences in the decrease of salivary cortisol levels [15].

However, natural environments may not always be restorative, despite the fact that people tend to recover more quickly from stress and mental fatigue in natural than in urban settings [16]. In fact, physical environment can heighten or ameliorate stress, while meteorological factors also cause a certain stress on the human body [17]. Changes in meteorological parameters may affect human hemodynamics and other factors adversely, thereby catalysing the event of an acute coronary syndrome [18]. To this extent, little is known about the restorative or exacerbating potential of natural environments for weather-sensitive (WS) individuals. Concerning reactions to weather variations, people can be divided into two main groups: weather-resistant and WS [19, 20]. The term “weather sensitivity” is used to define the impairment of well-being and/or incidence of symptoms or exacerbations of diseases in response to weather changes [21]. According to the results of our earlier study, almost 50% of individuals with CAD described themselves as being WS [22].

WS profile and the effects of walking in nature on the psychophysiological stress response in individuals with CAD (WE_SENSE_THE_NATURE) is a pioneer study, aiming to investigate how weather sensitivity and walking in different environments affects psychophysiological responses to the stress of individuals with CAD during rehabilitation. The proposed randomised control trial will provide insight on the influence of environmental exposure, as it is seldom investigated in association to heart disease.

Specifically, we aim to explore the link between personality traits and cognitive functioning (especially cognitive flexibility) with WS. Compulsive personality traits and distressed personality profile together represent risk factors that affects mental and physical health [1, 23–28]. Reduced cognitive functioning is also known to negatively impact many aspects of individual functioning, including the psychological, social, and occupational spheres [29, 30]. Studies investigating a set of cognitive, clinical, and personality predictors of functional outcomes of patients with CAD are therefore needed, but seldom investigated integratively. Thus our explorative aim is to understand associations between WS and personality characteristics as well as cognitive functioning in patients suffering from CAD. This exploration may reveal significant information which could help to stratify and apply treatment possibilities available for stress-vulnerable CAD patients. A patient-tailored approach which is timely and appropriate would be much welcome in the face of the rapidly unfolding climate change.

## Methods / design

### Aim

The aim of this study is to investigate how WS and walking in different environments affect psychophysiological responses to the stress of individuals with CAD during rehabilitation.

To achieve the main goal, we will aim to deliver a set of specific objectives:

1. Determining how walking outdoors (in a natural setting) affects the psychophysiological reactions to stress in individuals with CAD.
2. Determining how walking indoors (in the gym) affects the psychophysiological reactions to stress in individuals with CAD.
3. Investigating the associations between the psychophysiological reactions to stress of individuals with CAD and the walking environment, by accounting for their weather-sensitivity.
4. Understanding the connections between mental flexibility and weather sensitivity in the context of personality characteristics and mental distress.

We hypothesize that: (1) the psychophysiological reactions to stress in people with CAD will differ between the experimental (walking outdoors) and the control group (walking indoors); (2) for the experimental group, we hypothesize that psychophysiological reactions to stress will differ between WS individuals and non-WS individuals, showing more favorable results in the latter subgroup (non-WS individuals); (3) for the control group, we hypothesize that psychophysiological reactions to stress will differ between WS individuals and non-WS individuals, showing more favorable results in the former subgroup (WS individuals). The fourth goal is exploratory and therefore has no hypothesis.

### Study design, setting and participants

This randomised control trial protocol has been developed in accordance with the SPIRIT (Standard protocol items: recommendations for interventional trials) guidelines for protocol reporting [31]. The SPIRIT checklist is available as supporting information (see Supplementary Material 1).

The study will be performed at the Laboratory of Behavioral Medicine of Neuroscience Institute (NI) of the Lithuanian University of Health Sciences (LUHS) in Palanga, Lithuania. The individuals with CAD attending a rehabilitation program at the Palanga Clinic of the LUHS NI will be invited to participate in this study. Individuals who comply with the following inclusion criteria will be considered for inclusion in the study: (1) age 18 years and older, (2) diagnosis of CAD, (3) participation in the cardiac rehabilitation program, (4) ability to hear, speak and read in Lithuanian, and (5) signed informed consent. The selection of age ensures that the research specifically targets adults, since some reactions to treatments may differ across different age cohorts, particularly very young individuals. By including individuals who are actively engaged in a cardiac rehabilitation programme, the research will focus on a particular subgroup of the population with CAD who will receive an intervention aiming to improve standard rehabilitation practices. Requiring participants to hear, speak, and read in Lithuanian will ensure language consistency throughout the study and that the investigation is culturally appropriate and contextually relevant. A signed informed consent form will serve as documentation that participation in this particular study is voluntary.

Exclusion criteria will comprise (1) coronary artery bypass graft surgery, other cardiac surgery, (2) cognitive disabilities or other severe comorbidities, (3) unstable cardiovascular status. Focusing on individuals with CAD without the potentially confounding effects of recent surgical interventions will ensure a relatively stable subjects’ group. Cardiac surgeries can introduce variability in health status, recovery, and medication regimens, which may impact the study outcomes [30]. Individuals with cognitive dysfunction or severe comorbidities, especially in CAD [29], may introduce confounding factors that affect their ability to comprehend and adhere to the study protocol. Importantly, excluding individuals with unstable cardiovascular status will prioritise safety of those individuals who undergo rehabilitation and will align with ethical considerations.

### Study procedure

**Fig. 1:**
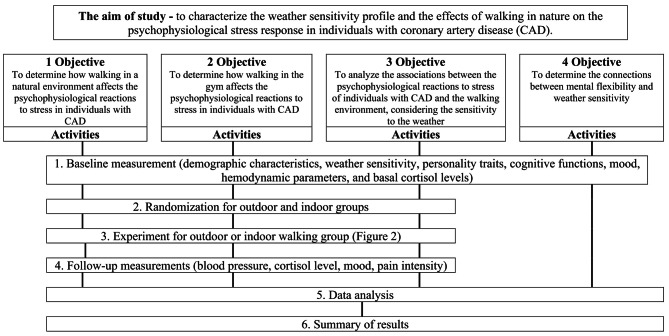
Study design: A breakdown structure depicting chronological steps (activities) to attain each of the predefined objectives

**Fig. 2:**
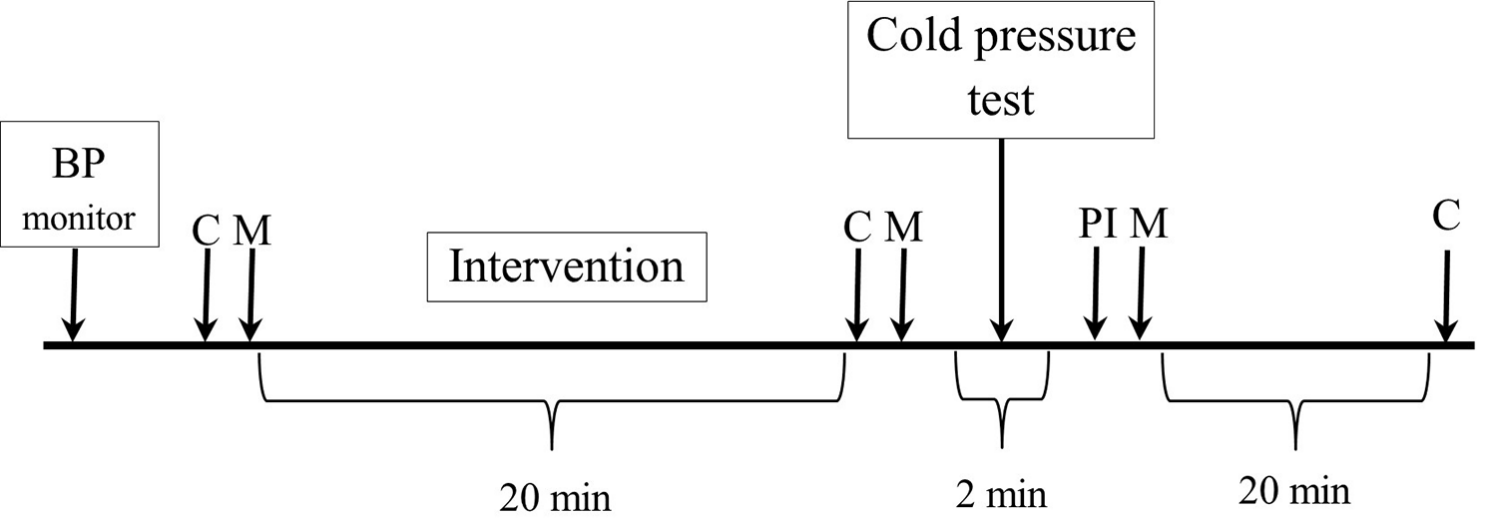
The experiment sequence of the study Note. BP monitor – continuous blood pressure measurement; C – saliva collection for cortisol analysis; M – mood; PI – pain intensity; Intervention – walking outdoor or indoor

The patients will be informed about the purpose and procedures of the study before signing the informed consent form. The breakdown structure of the study activities is presented in Fig. 1. A cardiologist will consult each patient about their eligibility to take part in the experiment, and their functional capacity will also be assessed. Following the consultation with the cardiologist, additional baseline assessments will be performed to evaluate demographic and clinical characteristics, weather sensitivity, Type D personality, subjective mental health status, and basal cortisol level. Basal cortisol level will be registered by testing cortisol concentration in saliva in the morning and in afternoon, one day prior to the experiment. After baseline measurements, patients will be randomly assigned to two groups: walking outdoors (OUT) or walking indoors (IN). OUT group will walk in the city park located within a 2–3 min walk of the LUHS NI Palanga Clinic along a pre-designated route. IN group will walk in a gym on a treadmill. The order and sequence of measurements within each trial will be the same (Fig. 2).

### Randomisation

Depending on the date and time of consent to take part in the study, every second individual will be consecutively assigned to one of the conditions (e.g., the first individual will walk outdoors, the second will walk indoors, the third will walk outdoors, a fourth will walk indoors again and so on) without any prior preference or prioritization whatsoever.

### Experiment procedure

On the day of the experiment (Fig. 2), we will evaluate the effect of walking in different environments on hemodynamic parameters, stress level (according to cortisol concentration in saliva), and mood. In the morning, the patient will be fitted with a long-term blood pressure device, which will be removed the next morning. In this way, the patient will also receive detailed information about their hemodynamics. Both a collection of saliva samples and a mood assessment will be performed immediately before and after the intervention (walking). A cold pressure test (short immersion of patient’s hand into a cold container) will then be administered, followed by a third mood assessment. The third saliva sample will be collected 20 min after the cold pressure test. Cortisol levels rise progressively after a few minutes (about 10 min) of stimulation and peak between 10 and 30 min after stress termination [32, 33].

Participants in the OUT group will be instructed to keep a comfortable walking speed and to enjoy the walk and the surroundings. Participants in the IN group will be introduced to the treadmill and encouraged to choose a walking speed like that of leisurely outdoor walking. All participants will be informed that they will be accompanied by the researcher (at a distance) during the intervention to monitor their walking time, minimize the impact of social interaction, and control any potential well-being or health problems.

### Measurements

A set of validated instruments are listed in Table 1 and will be used in this investigation. These tools have all been vvalidated and are often used in Lithuanian clinical and research settings. The research will involve a variety of actions and measures, as listed in Table 1.

**Table 1:** Steps and measures of the study

Step	Variable	Instrument	Measure	Procedure duration
1.	Weather sensitivity	Questionnaire (short form)	The Palanga Weather Sensitivity Self-Assessment Diary (PSAD-WS) will be completed to assess weather sensitivity [22]. PSAD-WS is an 11-item (general) three-factor tool for collecting information regarding weather sensitivity in patients with CAD. The three subscales reflected (1) psychological symptoms, (2) cardiac symptoms and (3) physical. Also, patients will be asked a single question: “Do you feel the weather changes?” with possible answers of “NO” or “YES”.	up to 2 min
2.	Cortisol level	Saliva samples will be obtained using “SaliCap Set“ releasing the saliva into the sampling tubes. Samples will then be stored at -70^o^C and cortisol levels will be determined in a licensed laboratory using commercial reagent kits.	To assess hypothalamic pituitary adrenocortial (HPA) axis activity, cortisol will be sampled and assayed from participants’ saliva.	up to 20 min
3.	Sociodemographic characteristics and clinical history	Medical records	Age, sex, education, medical history [i.e., New York Heart Association (NYHA) functional class, history of ACS, and angina pectoris class], and medication.	up to 15 min
4.	Functional capacity	The six-minute walk test (6MWT) [34]	The 6MWT will be performed in a 30 m hallway and will be monitored by a physiotherapist. The patient will instructed to remain calm, to have taken his/her medications and to wear comfortable clothing and shoes. All tests will be performed between 8:00 am and 12:00 am in a quiet indoor hallway with a flat straight floor. The floor will be marked in 1 m increments and total distance covered will be recorded. Participants will be asked to walk at their own pace, while attempting to cover as much ground as possible within the allotted six minutes. Every minute, a physiotherapist will encourage subjects to continue walking and inform them of the time elapsed, using standardized phrases (“You are doing well” or “Keep up the good work”). The patient’s heart rate, BP will be measured before and after the test. Any symptoms experienced by the patient during this test will also be recorded (Borg scale) [35]. The distance walked will be recorded after the test.	up to 10 min
5.	Mental health outcomes	Patient Health Questionnaire (PHQ) [36, 37]	The PHQ assesses 8 diagnoses, divided into threshold disorders (disorders that correspond to specific diagnoses in the Diagnostic and Statistical Manual of Mental Disorders, Fourth Edition, specifically major depressive disorder, panic disorder, other anxiety disorder, and bulimia nervosa) and subthreshold disorders (disorders for which criteria encompass fewer symptoms than are required for any specific diagnoses in Diagnostic and Statistical Manual of Mental Disorders, Fourth Edition, specifically other depressive disorder, probable alcohol abuse or dependence, and somatoform and binge eating disorders). This questionnaire will be mainly treated to control our findings for confounding mental health status.	up to 15 min
6.	Type D personality	Type D Personality Scale (DS14) [38]	The DS14 is a 14-item self-rating questionnaire that consists of two 7-item subscales that are designed to measure personality traits of negative affectivity (NA) and social inhibition (SI), Each item is rated on a five-point Likert-type scale from 0 (false) to 4 (true) with scores ranging from 0 to 28 on each subscale. Scores equal or greater than 10 on both DS14 subscales of NA and SI, indicate Type D personality.	Up to 5 min
7.	Compulsive Personality	Compulsive Personality Assessment Scale (CPAS) [39]	The CPAS includes 8 items and measures the severity of obsessive-compulsive personality traits and tendencies. Each of the eight diagnostic criteria is scored on a Likert scale of 0 to 4, and the maximum total score of the CPAS is 32. Diagnosis of obsessive-compulsive personality disorder can be defined as a score of three (severe) or four (very severe) on at least four of the CPAS items.	Up to 3 min
8.	Cognitive function	Cambridge Neuropsychological Test Automated Battery [40]	CANTAB will be used to evaluate alterations in executive function as follows: - Motor screening Task (MOT) – sensorimotor function. - Cambridge Gambling Test – decision making (impulsivity, risk taking) - Delayed Matching to Sample – short-term visual recognition memory; - Intra-Extra Dimensional Set Shift (IED) – set-shifting, mental flexibility; - Match to Sample Visual Search (MTS) – attention and visual searching; - One Touch Stockings (OTS) – spatial planning and the working memory; - Rapid Visual Information Processing (RVP) – sustained attention; - Spatial Working Memory (SWM) – working memory. Tests have been validated in behavioral and psychopharmacological studies on healthy volunteers and in a range of patient groups [41].	up to 60 min
9.	Hemodynamic parameters	A long-term blood pressure (BP) device – The SOMNOtouch NIBP monitor	The SOMNOtouch NIBP monitor will be used to make a simultaneous, continuous, and non-reactive recording of BP, ECGs, and oxygen saturation thus a comparative analysis of several vital parameters will be provided. A long-term BP monitor will be placed on the patient at the beginning of the study.	Up to 24 h
10.	Mood	Visual Analog Scales (VAS) [42]	Participants simply will rate the intensity of the sensation on a scale from 0–100. VAS were chosen based on its applicability in experimental clinical studies.	Up to 1 min
11.	Cold pressure stress	Cold pressure test (CPT) [43]	CPT will be used to induce acute stress. Temperature and other environmental stressors are known to affect BP and heart rate. In this activity, participants will be performed the cold pressure test to demonstrate the changes in BP that follow an environmental stress. For cold stress, the subject will immerse his or her right hand in the ice-water bath (4 °C) to a point just above the elbow for 2 min. BP measurements will be obtained using a long-term BP device – the SOMNOtouch NIBP monitor on the wrist of the left hand at 0, 1, 2, and 4 min after the right hand will be removed from the ice-water bath.	Up to 8 min
12.	Pain intensity	Visual Analog Scales (VAS) [42]	After the CPT, study participants will be debriefed about the purpose of the study and the subjective measure of perceived efforts and perceived difficulty of the CPT will be collected by using VAS. The scales ranged from 0 (maximum difficulty/efforts) to 100 (minimum difficulty/efforts). VAS were chosen based on its applicability in experimental clinical studies and common use in combination with CPT [44].	Up to 1 min

### Sample size

Based on previous research in this area [45], considering the cortisol response to psychophysiological stress as a primary approach to detect a likely medium difference between groups effect (Cohen d = 0.5) and achieve sufficient power1 (1-ß err prob = 0.95) requires a sample of at least *n* = 82 subjects. Considering possible side variable of weather sensitivity and differences in natural conditions, likelihood of 4% attrition, the sample is planned to be doubled to *N* = 164 subjects. The enrollment duration - one year.

### Statistical analysis

All statistical analyses will be conducted using the IBM SPSS Version29.0.0.0 (Chicago, IL, USA). Variable distribution of similarity to normal will be assessed visually and using the Kolmogorov-Smirnov and Shapiro‐Wilk tests. Student’s t‐test, Mann‐Whitney test, and/or Wilcoxon rank test will be used for data comparison between groups. General Linear Modelling will be used for multiple dependent variables comparisons. Frequency, dependence, and homogeneity of statistical significance will be tested using the Chi square test, or/and Fisher’s exact test. The probability of equality will be tested using z‐test. Binary, multivariate logistic, linear, and nonlinear regression will be used to test correlations of the study findings adjusting for possible confounders.

## Discussion

The proposed protocol provides a general overview of the research methodology of the study WE_SENSE_THE_NATURE. The purpose of this study is to increase the knowledge about the impact of the natural environment on well-being and health, providing more information to both health professionals and the public. We will conduct an experimental study including individuals with CAD attending cardiac rehabilitation. We hypothesize that psychophysiological reactions to stress in individuals with CAD will differ with respect to the walking environment (between OUT and IN group walking in nature vs. gym respectively) and weather sensitivity (between WS and non-WS individuals who experience a weather sensitivity or lack of weather sensitivity).

This randomized controlled trial will be the first to investigate the influence of walking in different environments on psychophysiological responses to stress of individuals with CAD during rehabilitation, and to evaluate the importance of the weather sensitivity profile. This research project might provide important insight on the importance of environmental exposure, as it is still an underappreciated risk factor contributing to the development and severity of heart disease.

Additionally, findings on personality traits and cognitive functions (especially cognitive flexibility) in relation to WS may reveal significant information which could help to even better stratify and individual treatment possibilities available for stress-vulnerable individuals with CAD. Weather sensitivity profiling in the context of personality type and individual’s cognitive flexibility might prove to be useful in adapting personalised regimen of physical activity in cardiac rehabilitation.

Understanding the nature of the environmental impact on the human body could aid in the preventive treatment of a broad spectrum of health disorders [20]. Despite significant advancements in prevention and control, CVDs continue to be a leading cause of health problems and deaths worldwide [21], including Lithuania [22]. CVD treatment requires a comprehensive approach owing to its significant effects on healthcare services. While traditional risk factors, such as smoking, alcohol use, hypertension, high cholesterol levels and obesity, are fundamental in explaining a substantial number of CVDs, numerous features of the environment have been discovered to have a significant impact on CVD risk, development, and course [18]. For instance, many studies have focused on the role of extreme temperatures in cardiovascular emergencies. Heat exposure has been linked to a higher risk of cardiovascular emergencies [46], although some authors have shown that this relationship is only present above a certain temperature threshold [47]. On the other hand, low temperatures have also been associated with higher CVD risk [48]. Other climatic factors may also influence cardiovascular health. Wind velocity, atmospheric pressure, and sunshine exposure have been linked to a higher incidence of myocardial infarction (MI) [49]. However, the exact role of many environmental variables in CVD remains unclear, with some studies showing contradictory results. Among these variables is precipitation, with some authors finding no consistent role of rain- or snowfall in the incidence of acute coronary syndromes [50], whereas others have demonstrated a relationship between snowfall and MI-related hospitalisation or death [51]. Such discrepancies could possibly result from varying weather conditions depending on the location of the study. Moreover, the snowfall-MI risk relationship has been shown to be stronger in men [50]. This highlights the necessity of considering both regional and individual variability when considering the role of weather in CVD. Therefore, assessment of the potential contribution of environmental factors to the course of CAD is important, as many such factors are modifiable either through individual behaviors or government regulation, and early intervention has the potential for significant public health benefits [48].

Rehabilitation is one of the main aspects included in CVD medical care. Cardiac rehabilitation programs aim to reduce the risk of another heart event, to monitor and control the current heart condition, and to improve the health and quality of life of patients with CVDs [7]. A cardiovascular rehabilitation program includes standard secondary CVD prevention according to current guidelines, including physical therapy with daily outdoor walks [7].

To date, there are no clear pathophysiological links between weather and cardiovascular diseases. Climatic stress may increase direct and indirect risks to human health via different, complex pathophysiological pathways and exogenous and endogenous mechanisms [52]. Heat may compromise the cardiovascular system by causing dehydration, vasodilation, changes in coagulation, and damage to the vascular endothelium, which may ultimately result in an autonomic imbalance and pose a risk of hemorrhage and thrombosis [46]. On the other hand, cold exposure can pose a risk to the cardiovascular system by causing a rise in myocardial oxygen demand, increasing cholesterol build-up in atherosclerotic plaques, and promoting hypercoagulability [48]. This duality may explain why many authors have reported a non-linear, often U-shaped, relationship between temperature and cardiovascular mortality [2]. Therefore, both extremely cold and extremely hot conditions can pose a threat to cardiovascular health. The unfolding of climate change comes with a bigger range of temperature oscillations and more extreme weather events and might therefore pose a tangible risk for CAD patients.

Awareness about the potential influence of weather sensitivity on the psychophysiological reactions to stress in patients with CAD is much needed as it may contribute to a timely planning and implementation of actions leading for improved medical care services and preventive measures, in the face of the expected climate change. This in turn, would help to avoid the worsening of health and well-being in the future. Protective measures should be directed towards susceptible groups rather than the entire population. The outcomes of this experiment may have direct contribution to three fields:

- clinical practice - using different types of environments in cardiac rehabilitation. If the hypotheses are confirmed, for weather-sensitive patients with CAD, it might be better to engage in physical activity in an indoor environment than in an outdoor one;
- sciences and education. Whilst the study will be carried out in Lithuania, it is hoped that the methods used and the results obtained will contribute to a better understanding of the problem analyses and will be useful for future insights and research not only in Lithuania, but also on an international level. It might also provide a foundation for new research studies looking into and beyond traditional risk factors identifying high-risk subgroups of patients with CAD vulnerable to stress.
- health services’ management - to the government and policymakers in planning and implementing actions to improve medical care services in rehabilitation and post-rehabilitation settings.

### Strengths of the study

- This research offers a more integrative approach to understanding of the processes that contribute the psychophysiological stress response in CAD patients, by accounting for both the dispositional and the situational factors.
- The study could provide deeper insight the effects of walking in nature (vs. gym) on the psychophysiological stress response in individuals with CAD, by accounting for their sensitivity to weather (and profiling them accordingly), as well as a set of other parameters.

### Limitation

Participant recruitment from a single cardiac rehabilitation centre may have subject our results to selection bias. Additionally, it might lead to a prolonged recruitment procedure (lasting up to one year), in order to obtain optimal sample size.

We will measure cortisol levels during early recovery period which will preclude us from reporting the data on cortisol variability during late recovery periods (for example next day measures).

Finally, we consider this as a pilot study, thus similar replication studies are warranted for future research to guide the recommendation process.

### Electronic supplementary material

Below is the link to the electronic supplementary material.

Supplementary Material 1

## Data Availability

No datasets were generated or analysed during the current study.
